# High prevalence of trypanosomes in European badgers detected using ITS-PCR

**DOI:** 10.1186/s13071-015-1088-7

**Published:** 2015-09-22

**Authors:** Eze J. Ideozu, Andrew M. Whiteoak, Alexandra J. Tomlinson, Andrew Robertson, Richard J. Delahay, Geoff Hide

**Affiliations:** Ecosystems and Environment Research Centre, School of Environment and Life Sciences, University of Salford, Salford, M5 4WT UK; National Wildlife Management Centre, Animal and Plant Health Agency, Woodchester Park, Gloucestershire, GL10 3UJ UK; Environment and Sustainability Institute, University of Exeter, Penryn, Cornwall, TR10 9EZ UK; Biomedical Research Centre, School of Environment and Life Sciences, University of Salford, Salford, M5 4WT UK

**Keywords:** *Trypanosoma*, Badger, *Meles meles*, PCR, Ribosomal RNA genes, ITS-PCR

## Abstract

**Background:**

Wildlife can be important sources and reservoirs for pathogens. Trypanosome infections are common in many mammalian species, and are pathogenic in some. Molecular detection tools were used to measure trypanosome prevalence in a well-studied population of wild European badgers (*Meles meles*).

**Findings:**

A nested ITS-PCR system, that targeted the ribosomal RNA gene locus, has been widely used to detect pathogenic human and animal trypanosomes in domestic animals in Africa and some wildlife hosts. Samples from a long-term DEFRA funded capture-mark-recapture study of wild badgers at Woodchester Park (Gloucestershire, SW England) were investigated for trypanosome prevalence. A total of 82 badger blood samples were examined by nested ITS-PCR. Twenty-nine of the samples were found to be positive for trypanosomes giving a prevalence of 35.4 % (25.9 % - 46.2 %; 95 % CI). Infection was not found to be linked to badger condition, sex or age. Analysis of DNA sequence data showed the badgers to be infected with *Trypanosoma (Megatrypanum) pestanai* and phylogenetic analysis showed the Woodchester badger trypanosomes and *T. pestanai* to cluster in the *Megatrypanum* clade.

**Conclusions:**

The results show that the ITS Nested PCR is an effective tool for diagnosing trypanosome infection in badgers and suggests that it could be widely used in wildlife species with unknown trypanosomes or mixed infections. The relatively high prevalence observed in these badgers raises the possibility that a significant proportion of UK badgers are naturally infected with trypanosomes.

**Electronic supplementary material:**

The online version of this article (doi:10.1186/s13071-015-1088-7) contains supplementary material, which is available to authorized users.

## Findings

### Background

Trypanosomes are blood parasites of vertebrates with several species being agents of disease in humans and/or animal populations [1]. They are found globally, including the UK, and infect a wide range of species. To date, a variety of trypanosome species have been detected in British fauna, with most being from the subgenera *Herpetosoma* and *Megatrypanum* [2].

The European badger (*Meles meles*) is a widespread and common wild mammal in the UK. Studies conducted on British badgers in Cornwall [3] and Wytham Woods, Oxfordshire [2, 4] have shown them to be infected with *Trypanosoma (Megatrypanum) pestanai* but knowledge about infection of badgers with trypanosomes in other geographical areas is sparse. To date, the only study using molecular tools for the determination of prevalence of infection has been the Wytham Woods study that used an 18S gene PCR to determine the prevalence of *T. pestanai* as 31 % in the badger population [2]. Although, there are many molecular methods useful for the detection of trypanosomes [5] in humans and domestic animals, one method that can be used on a wide range of trypanosome species is the Internal Transcribed Spacer (ITS) Nested PCR based on generic trypanosome primers [6–8]. The technique is capable of distinguishing between various species of trypanosomes by amplification of the variable length Internal Transcribed Spacer region of ribosomal RNA and producing unique band sizes for each species/subspecies of trypanosome. The absence of DNA sequence data for the full ITS-rRNA in the databases for many trypanosome species of wildlife limits *in silico* approaches to determining ITS-Spacer size for many trypanosome species. 18S rRNA sequence data and concatenation of 18S and GAPDH sequences have been widely used for phylogenetic analysis [1, 9–11], but little is known about the phylogenies of trypanosomes generated using 28S rRNA genes. This is partly due to the lack of availability of many trypanosome 28S rRNA sequences including that of *Trypanosoma pestanai*.

The primary objective of this study was to investigate the prevalence of trypanosome infections using the ITS Nested-PCR on blood samples from badgers in the well-studied population at Woodchester Park in Gloucestershire. A secondary objective was to derive the 28S rRNA sequence from *T. pestanai* and apply molecular phylogenetic analyses using this marker to compare with the 18S phylogenies.

## Methods

Blood samples were obtained from 82 badgers at Woodchester Park, Gloucestershire, UK as part of an ongoing long term capture-mark-recapture study [12, 13]. Badgers were live-trapped in steel mesh box traps baited with peanuts, and subsequently examined under anaesthesia before being released at the point of capture. At each capture event, data was collected on the age, gender, location of capture of the animal and biometric measurements were taken. A blood sample was taken from each captured animal and stored at −20 °C before being processed in the laboratory. All badgers in the present study were captured during a single summer season from May to June 2012 inclusive and hence there are no seasonal effects such as variation in body condition. For each badger, the scaled mass index (SMI) - a modification of the condition index [14, 15] - was calculated. The SMI values were calculated using scaling coefficients and mean body length derived from 5059 adult female, 2157 adult male and 2303 cub capture events taken from the Woodchester Park historic database. As the SMIs for the adult males, adult females and cubs differed, each value was standardised by subtracting the mean and dividing by the standard deviation. Badgers with an SMI of <1 indicated a poorer condition while SMI of >1 indicated a better condition.

DNA was extracted using a modification of a phenol-chloroform protocol as described previously with appropriate measures to prevent contamination [16]. Extracted DNA was tested for its ability to amplify by PCR using a set of mammalian tubulin PCR primers, as previously described [16]. Extracted DNA from badger blood was tested for trypanosome infection using the ITS-Specific (nested) PCR method following standard protocols [7]. PCR Primers and reaction conditions used to amplify the 18S region of *T. pestanai* are described in Additional file 1: Table S1. The 28S ribosomal RNA of *T. pestanai* was a target region for PCR amplification however the appropriate 28S sequence was not available in the databases. Suitable primers for novel amplification of the 28S rRNA gene from *T. pestanai* were designed using genes from closely related trypanosomes (see Additional file 2: Table S2). PCR products and their associated primers were outsourced to a sequencing company (Source Bioscience) for purification and sequencing.

The Fisher’s Exact Test and Chi-Square tests were used to assess the association between categorical variables, the Odds ratio (OR) and 95 % Confidence Intervals (CI). For investigating the relationship between the Standardised Scaled Mass Index (SSMI) and trypanosome infection, badgers were allocated to one of four body condition categories (Very Poor, SSMI < −0.75; Poor, SSMI −0.749 to 0.0; Fair, SSMI 0.01 to 0.75; Good, SSMI >0.751) and associations tested as above.

Molecular phylogenetic trees were constructed using parameters described in Additional file 3: Table S3.

For ethical approval, all animal procedures on the badgers were covered by licences issued by the Home Office and the Veterinary Medicines Directorate, following approval by ethics panels at The Food and Environment Research Agency and Animal Health and Veterinary Laboratories Agency. The study was conducted according to the principles of Good Clinical Practice.

## Results and Discussion

A total of 82 badger blood samples were tested for trypanosome infection using the ITS-Nested PCR method [7]. Twenty-nine out of the 82 DNA samples amplified using ITS-Nested PCR producing band sizes of approximately 1271 bp indicating the badgers were positive for trypanosomes. This gives a prevalence of 35.4 % (25.9 %–46.2 %; 95 % CI). The 18S PCR was carried out on badger DNA samples that were positive for trypanosome DNA, to identify the species detected and confirm that the ITS-PCR technique was indeed detecting the target species (*T. pestanai*). The novel 28S region was successfully amplified, producing a band size of 2460 bp, and sequenced [Accession Number KR527480]. No variation was seen between badger trypanosome samples using this marker.

**Fig. 1:**
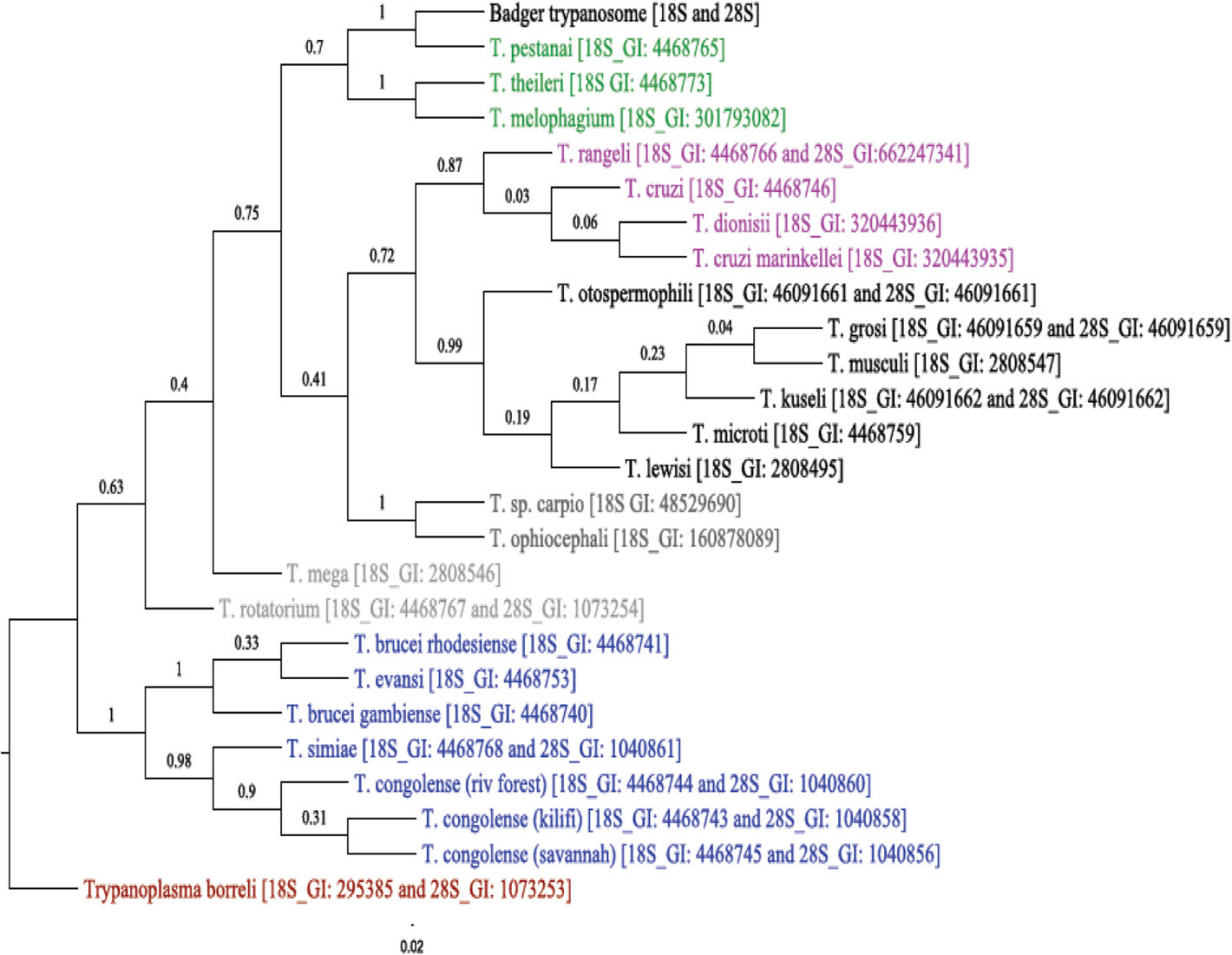
Phylogenetic tree of concatenated SSU-rRNA and LSU-rRNA sequences of badger trypanosomes and comparator species. The phylogenetic analysis was implemented using the Maximum Likelihood method based on the Kimura 2-parameter model. The tree with the highest log likelihood (−4361) is shown. Initial trees for the heuristic search were obtained automatically by applying Neighbor-Join and BioNJ algorithms to a matrix of pairwise distances estimated using the Maximum Composite Likelihood (MCL) approach, and then selecting the topology with the superior log likelihood value. A discrete Gamma distribution was used to model evolutionary rate differences among sites (5 categories (+G, parameter = 0.1323)). The analysis involved 26 nucleotide sequences, 11 of which, used the concatenated datasets (*T. grosi, T. otospermophili, T. kuseli, T. rangeli, T.pestanai, T. rotatorium, T. simiae, T. congolense (riverine forest), T. congolense (kilifi), T. congolense (savannah), Trypanoplasma borreli* and badger trypanosome). There were a total of 1560 positions in the final dataset. The numbers after species name on branch are the GenInfo Identifier number (GI) while annotated colours indicate different groups of kinetoplastids. Evolutionary analyses were conducted in MEGA6.  *Herpetosoma*  *Schizotrypanum*  *Megatrypanum*  Fish trypanosomes  Amphibian trypanosomes  Salivarian trypanosomes  Bodonid

In order to investigate whether the distribution of infected animals (35.4 % prevalence) was evenly spread throughout the Woodchester Park study area, the prevalence of trypanosome infection was examined by gender, body condition and social group. Although, more females (55.2 %) were infected than males (44.8 %), there was no significant association between trypanosome infection and sex (*p* = 0.771)). Using the Standardised Scale Mass Index (SSMI), badgers were categorised into groups based on relative body condition status (very poor, poor, fair, good–see Methods). There was no relationship between body condition and prevalence of *T. pestanai* infection in the badgers (*p* = 0.80). Furthermore, there was no significant difference in the prevalence of infection between adults and cubs (*p* = 0.98). Badgers used in this study were captured from 26 different social groups (Table 1) and *T. pestanai* was detected in 15 of those (58 %, Table 1). However, there was no significant association between badger social group and trypanosome infection prevalence (*p* = 0.464). As the sample size for each social group was small, they were pooled into different regions (East and West; North and South) to investigate any broader geographical differences. Fifty-two (63.4 %) of the badgers were captured in the north, of these, 18 were infected resulting in 34.6 % prevalence while the remaining 30 (36.6 %) were captured in the south and 11 were infected resulting in 36.7 % prevalence. The odds of being a prevalent case was slightly higher for badgers captured in the south (OR = 1.094; 95 %; CI: 0.428–2.791) although there was no significant difference (*p* = 1.0). Additional analysis carried out in the remaining two quadrants (East and West) of the study area showed that of the 29 (35.4 %) badgers captured in the east, 12 were infected (41.4 % prevalence) while from the remaining 53 (64.6 %) captured in the west 17 were infected (32.1 % prevalence). Although, the odds of infection with trypanosomes was reduced for badgers captured in the East (OR = 0.669; CI: 0.262–1.708) than those in the West the reduction was statistically not significant (*p* = 0.472). To investigate the phylogenetic relationship of *T. pestanai* with other trypanosome species, two sets of trees were generated. Firstly, a tree based on existing 18S (SSU-rRNA) sequences was constructed (data not shown) for comparison with a concatenated tree (Fig. 1) with the new 28S sequence (i.e. 18S _ 28S; SSU-rRNA + LSU-rRNA). *Trypanoplasma borreli* was used to form an out-group and its clade can easily be distinguished from the genus *Trypanosoma* in both the SSU-rRNA (data not shown) and concatenated tree (Fig. 1). The trypanosomes were divided into two major clades, one representing the salivarian trypanosomes while the other represented the stercorarian trypanosomes with the division being obvious in both the SSU-rRNA and concatenated trees. The *Megatrypanum* subgenus, appear to cluster together with a high bootstrap support values of 80 % on the SSU-rRNA tree and 70 % on the concatenated tree. Analysis from both trees showed that the badger trypanosome sequences determined from the Woodchester badgers, were positioned in the *Megatrypanum* clade and clustered together with the published *T. pestanai* sequences with a high bootstrap value of 100 % on both trees. This confirmed their identity as *T. pestanai* rather than any other closely related species.

**Table 1:** Social group of badgers and infection with trypanosomes

Social group at time of capture		Badger population frequency		Trypanosome infection status		Total
		Frequency	Percent	Not infected	Infected	
Arthurs		2	2.4	2	0	2
Beech		7	8.5	4	3	7
Breakheart		1	1.2	1	0	1
Boxwood		1	1.2	1	0	1
Cedar		5	6.1	2	3	5
Colepark		1	1.2	1	0	1
Colliers wood		1	1.2	1	0	1
Honeywell		11	13.4	8	3	11
Inchbrook		5	6.1	4	1	5
Jacks		4	4.9	2	2	4
Junction		4	4.9	2	2	4
Kennel		3	3.7	1	2	3
Larch		6	7.3	5	1	6
Mead		1	1.2	0	1	1
Old oak		4	4.9	2	2	4
Parkmill		5	6.1	2	3	5
Scotland bank		2	2.4	0	2	2
West		5	6.1	5	0	5
Windsor edge		5	6.1	3	2	5
Woodfarm		1	1.2	1	0	1
Woodrush		1	1.2	0	1	1
Wychelm		2	2.4	2	0	2
Yew		2	2.4	2	0	2
Septic		1	1.2	1	0	1
Top		1	1.2	1	0	1
Nettle		1	1.2	0	1	1
Total	26	82	100	53	29	82

This study reported a 35.4 % prevalence of *T. pestanai* infection in badgers. This compares with previous studies on trypanosome prevalence in British badgers from Cornwall [3] and Wytham Woods, Oxfordshire [2, 4], with at 10 %, 7.7 % and 31 % respectively. Estimating an absolute prevalence rate may depend on the sensitivity of the diagnostic measure applied to detect trypanosomes in a sample population. For example, the studies that were based on detecting trypanosomes in blood smears from British badgers [3, 4] recorded the lower prevalence estimates (10 % and 7.7 % respectively) and this may be attributed to the difficulty in detecting trypanosomes in blood smears especially when the level of parasitaemia is very low. While a direct comparison cannot be made, the two studies using PCR based methods [2, this study] showed a higher prevalence than those using microscopical analysis [3, 4] which is consistent with (although not indicative of) a higher degree of sensitivity using the molecular approaches. The PCR based study [2], from the Wytham Woods population estimated prevalence to be 31 % (25.6 to 37.1; 95 % CI) which was not significantly different to that derived from the present study at Woodchester Park of 35.4 % (25.9 %–46.2 %; 95 % CI). The ITS-Nested PCR shows a comparable prevalence to the 18S PCR study [2] possibly suggesting that both methods have similar levels of sensitivity. However, the ITS-PCR method has the added advantage of the potential to be used in any mammalian wildlife species and potentially to detect any trypanosome species or species mixtures, thus, offering a more broadly useful tool for surveys of infection in wildlife.

However, despite the potential of the ITS Nested PCR [7] for generic use in wildlife species, sequencing of the 18S rRNA gene, available on databases, needs to be used initially to confirm the trypanosome species or identify possible new species. On the SSU-rRNA phylogenetic tree, the badger trypanosomes detected in this study clustered with *T. pestanai* (100 % bootstrap value), confirming their identity, and were associated with the *Megatrypanum* clade. However, the addition of a 28S rRNA sequence of the badger trypanosome did not improve the strength of the *Megatrypanum* clade association and neither did we observe improvement on any of the other major clades despite previous studies indicating that concatenation of multiple sequences improves phylogenetic inference [17].

## Conclusions

In conclusion, the use of nested ITS-PCR for the detection of *T. pestanai* in badgers shows that it has potential to be an effective tool for molecular detection and identification of trypanosomes in wildlife species such as badgers. Although mixed species infections were not detected in these samples, in the generic case of trypanosome detection in wild animals, unlike the 18S PCR, this approach offers the potential to identify such mixtures if present. Such mixtures have been identified in African cattle, pigs and wild animals [8, 18–20]. The observed prevalence of 35.4 % in this study and comparable figures from related studies [2] suggest that a significant proportion of UK badgers could be infected with trypanosomes. As a wide range of wildlife hosts potentially may carry trypanosome infections, perhaps of a variety of different species, the ITS nested PCR method offers a good initial approach to investigating these infections in previously unstudied wildlife hosts.

## Additional files

Additional file 1: Table S1. PCR Reaction Conditions for amplification of the 18S and 28S genes from *Trypanosoma pestanai. (DOCX 11 kb)*
Additional file 2: Table S2. Trypanosome 28S sequences used to generate the primers for amplifying the *T. pestanai* 28S gene. (DOCX 11 kb) Additional file 3:
**Construction of Phylogenetic Trees.** (DOCX 16 kb)
