# Supplementary material for: High prevalence of trypanosomes in European badgers detected using ITS-PCR
Source: Parasit Vectors. 2015 Sep 22;8:480. doi: 10.1186/s13071-015-1088-7 (PMC4580359; doi:10.1186/s13071-015-1088-7)
Supplement: Additional file 1: Table S1. — PCR Reaction Conditions for amplification of the 18S and 28S genes from Trypanosoma pestanai. (DOCX 11 kb) [file 13071_2015_1088_MOESM1_ESM.docx]

Additional file 1: Table S1. PCR Reaction Conditions for amplification of the 18S and 28S genes from *Trypanosoma pestanai*

| PCR Reaction | Primers | PCR Reaction Conditions |
| --- | --- | --- |
| 18S PCR | 18SFor 5’-CATGCATGCCTCAGAATCACTG-3’  18SRev 5’-CTGTTGCCCAAAATCTCACCTTGC-3’ | 24 µl reaction mixture for the PCR: MyTaq^TM^HS Mix (Bioline, London) and 5 µM each of forward and reverse primers. Thermocycling conditions: 1 min at 95^o^C, 35 cycles of 15s at 95^o^C, 15s at 56^o^C, 1.30 min at 72^o^C ; one cycle of 5 min at 72^o^C |
| 28S PCR | 28S1F 5'-CAGACCTGAGTGTGGCAGG-3'  28S1R 5'-AAGAAAGCTCACCGTGGGAGG-3' | 24 µl reaction mixture for the PCR: MyTaq^TM^HS Mix (Bioline, London) and 5 µM each of forward and reverse primers. Thermocycling conditions: 1 min at 95^o^C, 35 cycles of 15s at 95^o^C, 15s at 54^o^C, 1.30 min at 72^o^C; one cycle of 5 min at 72^o^C |
