# Supplementary material for: High prevalence of trypanosomes in European badgers detected using ITS-PCR
Source: Parasit Vectors. 2015 Sep 22;8:480. doi: 10.1186/s13071-015-1088-7 (PMC4580359; doi:10.1186/s13071-015-1088-7)
Supplement: Additional file 2: Table S2. — Trypanosome 28S sequences used to generate the primers for amplifying the T. pestanai 28S gene. (DOCX 11 kb) [file 13071_2015_1088_MOESM2_ESM.docx]

Additional file 2: Table S2. Trypanosome 28S sequences used to generate the primers for amplifying the *T. pestanai* 28S gene

| Trypanosome species | 28S Gene accession number |
| --- | --- |
| *T. otospermophili* | GI: 46091661 |
| *T. kuseli* | GI: 46091662 |
| *T. rangeli* | GI: 662247341 |
| *T. minasense* | GI: 159157536 |
|  |  |
