# Supplementary material for: High prevalence of trypanosomes in European badgers detected using ITS-PCR
Source: Parasit Vectors. 2015 Sep 22;8:480. doi: 10.1186/s13071-015-1088-7 (PMC4580359; doi:10.1186/s13071-015-1088-7)
Supplement: Additional file 3: — Construction of Phylogenetic Trees. (DOCX 16 kb) [file 13071_2015_1088_MOESM3_ESM.docx]

Additional file 3: Table S3.Construction of Phylogenetic Trees.

| Protocol for phylogenetic tree construction |
| --- |
| To conduct molecular phylogenetic analyses, the SSU-rRNA DNA sequences of 25 kinetoplastids were assembled from GenBank on NCBI database (http://www.ncbi.nlm.nih.gov/genbank/), including available LSU-rRNA sequences for 10 of the kinetoplastids. Also, the badger trypanosome SSU-rRNA and LSU-rRNA sequences derived from this study were included in the analysis. Twenty-four of the kinetoplastids were members of the Trypanosomatidae family, of the genus *Trypanosoma* while the remainder (to be used as an out-group) was a member of Bodonidae family. In the first phylogenetic tree, the SSU-rRNA sequences were aligned with Muscle (v3.7) [1] and poorly aligned sequences were removed using Gblocks, which is shown to make alignments more appropriate for phylogenetic reconstruction [2]. This was performed on the Phylogeny.fr webserver [3] before the selected suitable alignment was then uploaded to MEGA v. 6 [4], where the best-fit substitution model was determined and also specified to reconstruct the phylogenetic tree based on maximum likelihood using 1000 bootstrap replicates. The second phylogenetic tree was reconstructed using LSU-rRNA sequences of nine trypanosomes; *T. grosi* [GI: 46091659], *T. otospermophili* [GI:46091661], *T. kuseli* [GI:46091662], *T. rangeli* [662247341], *T. rotatorium* [GI: 1073254], *T. simiae* [GI:1040861], *T. congolense (riverine forest)* [GI: 1040860], *T. congolense (Kilifi)* [GI:1040858] and *T. congolense (savannah)* [GI: 1040856] including the LSU-rRNA of *Trypanoplasma borreli* [GI:1073253] and the Badger LSU-rRNA trypanosome sequence (TP28SRNA) derived from this study [Accession Number: KR527480]. Using the procedure described above, all LSU-rRNA sequences were aligned and ambiguous regions were removed before the alignments were added to their SSU-rRNA counterparts to derive a concatenated sequence dataset for the respective trypanosome species. Using concatenated data sets for phylogenetic reconstruction has been shown to increase the probability of phylogenetic accuracy [5]. Also, the alignments were uploaded in MEGA v. 6 [4], where the best-fit substitution model was selected and the phylogenetic tree was reconstructed based on maximum likelihood using 1000 bootstrap replicates. |

1. Edgar RC.MUSCLE: a multiple sequence alignment method with reduced time and space complexity. BMC Bioinformatics. 2004; 5:113.
2. Castresana J.Selection of conserved blocks from multiple alignments for their use in phylogenetic analysis. MolBiolEvol 2000; 17:540-52.
3. Dereeper A, Guignon V, Blanc G, Audic S, Buffet S, Chevenet F et al. Phylogeny.fr: robust phylogenetic analysis for the non-specialist. Nucleic Acids Res 2008; 36:W465-9.
4. Tamura K, Stecher G, Peterson D, Filipski A, Kumar S. MEGA6: Molecular Evolutionary Genetics Analysis version 6.0. MolBiolEvol 2013; 30:2725-9.
5. Gadagkar SR, Rosenberg MS, Kumar S.Inferring species phylogenies from multiple genes: concatenated sequence tree versus consensus gene tree. J ExpZool B Mol Dev Evol2005; 304:64-74.
